# CD9 expression rivals IDH mutation as a prognostic marker in glioma: a novel nomogram approach

**DOI:** 10.3389/fneur.2025.1507443

**Published:** 2025-04-30

**Authors:** Yue Peng, Qiang Ji, Xiangyu Guo, Weichunbai Zhang, Zixuan Yang, Wenbin Li

**Affiliations:** ^1^Department of Neuro-Oncology, Cancer Center, Beijing Tiantan Hospital, Capital Medical University, Beijing, China; ^2^National Institute for Data Science in Health and Medicine, Capital Medical University, Beijing, China

**Keywords:** Glioma, CD9, Cox proportional hazards regression, Mendelian randomization, Single-cell analysis

## Abstract

**Purpose:**

Glioma remains a lethal malignancy with limited prognostic biomarkers. This study evaluates the prognostic significance and functional mechanisms of tetraspanin CD9 in glioma to establish its clinical relevance and identify therapeutic strategies.

**Methods:**

Multi-omics analyses were performed using 1,033 glioma samples from TCGA and CGGA cohorts. A CD9-integrated nomogram was developed via Cox regression. Two-sample Mendelian randomization (MR) assessed the causal link between CD9 expression and glioma risk using IVW, MR-Egger, and WM methods. KEGG enrichment analysis identified biological pathways. Single-cell RNA sequencing from 20 glioblastoma cases was analyzed using CellChat and Monocle3 to explore CD9-mediated cell communication and lineage transitions. Protein-protein interaction (PPI) networks were constructed via STRING and GeneMANIA, and drug predictions were performed using DsigDB.

**Results:**

CD9 overexpression was an independent predictor of poor survival (HR = 1.28, 95% CI 1.03–1.58). The CD9-based nomogram showed high prognostic accuracy (CGGA: C-index = 0.805 ± 0.01, TCGA: C-index = 0.859 ± 0.02). MR confirmed a causal association between CD9 and glioma risk (IVW OR = 1.33, *p* < 0.05) with no horizontal pleiotropy. CD9 regulated glioma progression via calcium signaling and synaptic pathways, interacting with ITGB1 and CD81. Single-cell analysis revealed CD9-driven NPC-to-OPC transdifferentiation, linked to tumor proliferation. Emetine was identified as a potential CD9-targeting drug.

**Conclusion:**

CD9 is a prognostic biomarker in glioma, with causal evidence linking its overexpression to tumor development. Its integration into risk models enhances prognostic precision, while drug screening highlights emetine as a potential therapy.

## 1 Introduction

Primary central nervous system (CNS) tumors account for approximately 1.6% of all cancers (1), with gliomas representing 25.1% of primary brain tumors and 80.8% of malignant CNS neoplasms (2). The 2021 WHO classification (3) categorizes gliomas into astrocytomas, oligodendrogliomas, and glioblastomas, with molecular subtyping further stratifying prognosis: low-grade gliomas exhibit a median survival of 7 years (4), whereas glioblastomas demonstrate a dismal median overall survival of 14 months (5). Despite prolonged survival in low-grade cases, inevitable progression to high-grade disease persists (6). While therapeutic advances have modestly improved outcomes, accurate assessment of natural tumor behavior remains challenging due to universal treatment interventions. Recent shifts toward molecular classification (7) have redefined histologically low-grade but IDH-wildtype tumors as glioblastomas, underscoring the critical role of biomarkers in elucidating glioma biology (1). Key discoveries include glioma stem cells with self-renewal capacity (8) and profound inter−/intra-tumoral heterogeneity (9). Established biomarkers such as MGMT promoter methylation, 1p/19q co-deletion, IDH mutations, and EGFR amplification guide clinical decisions (10), while stemness markers like CD133 and ALDH1 highlight therapeutic resistance mechanism (11). However, limitations in predictive accuracy and standardization necessitate novel biomarkers to refine prognostic frameworks.

CD9, a member of the tetraspanin family, is a cell surface glycoprotein characterized by four transmembrane domains. This unique structural configuration enables CD9 to interact with multiple transmembrane receptors, forming multiprotein complexes that regulate diverse cellular processes such as cell fusion, adhesion, and motility (12). CD9 plays a pivotal role in intercellular communication and signaling networks. Rather than functioning as a cell surface receptor itself, CD9 acts as an organizer of multimolecular complexes, which include integrins, immunoglobulin superfamily members, heparin-binding epidermal growth factor-like growth factor, claudin-1, and other tetraspanin (13). CD9 has been implicated in critical biological processes such as cell adhesion and migration (14), cancer progression and metastasis (14, 15), platelet activation and aggregation (16), sperm-egg fusion during mammalian fertilization (17, 18), humoral immune responses (19), allergic reactions (20), and viral replication of HIV-1 and influenza (21). This study aims to establish CD9 as a prognostic biomarker for glioma through integrative multi-omics analysis, delineate its interaction networks driving tumor progression, and identify therapeutic agents targeting CD9-associated pathways. By bridging molecular insights with clinical translation, we propose a novel prognostic model and repurposed therapeutics to address unmet needs in glioma management.

## 2 Materials and methods

Clinical and genetic data of glioma patients were collected from the Chinese Glioma Genome Atlas (CGGA) (22) and The Cancer Genome Atlas (TCGA) databases. Following quality validation, 1,328 glioma patients with Radiotherapy, Chemotherapy, WHO grade, IDH mutation status, MGMT promoter methylation, 1p/19q codeletion status, age, gender, and CD9 expression were included. The patient inclusion and exclusion flowchart is provided in Supplementary Figure 1. Genetic data were preprocessed, and the optimal cutoff value for CD9 was determined using the maximum selection rank statistics method. The correlation between the CD9 gene and glioma prognosis was determined based on Kaplan–Meier (KM) survival curves. Univariate Cox regression analysis was performed using the CGGA database, with variables showing *p* < 0.05 and meeting the PH assumption considered as glioma prognosis-related factors. These factors were subsequently incorporated into a multivariate Cox proportional hazards model to estimate the partial regression coefficients (*β*) for each variable, and patient survival probabilities were calculated based on the model. Clinical and molecular features were integrated to construct a nomogram. Calibration curves were plotted to assess the agreement between predicted and observed survival events. The discriminative ability of the model was validated using TCGA as an independent dataset.

The genome-wide association study (GWAS) summary statistics for glioma were obtained from the FinnGen database.^1^ Genetic associations between CD9 and single nucleotide polymorphisms (SNPs) were derived from the eQTLGen consortium.^2^ All summary-level data were de-identified, and no additional ethical approval was required. Instrumental variables (IVs) were selected by identifying SNPs significantly associated with CD9 and glioma at a genome-wide significance threshold (*p* < 5 × 10^–8^). Candidate SNPs were further subjected to linkage disequilibrium (LD) analysis to exclude weak instruments (r^2^ < 0.001 within a 10,000 kb window). Exposure and outcome datasets were harmonized to align allele effects, and weakly palindromic SNPs were removed.

**Figure 1:**
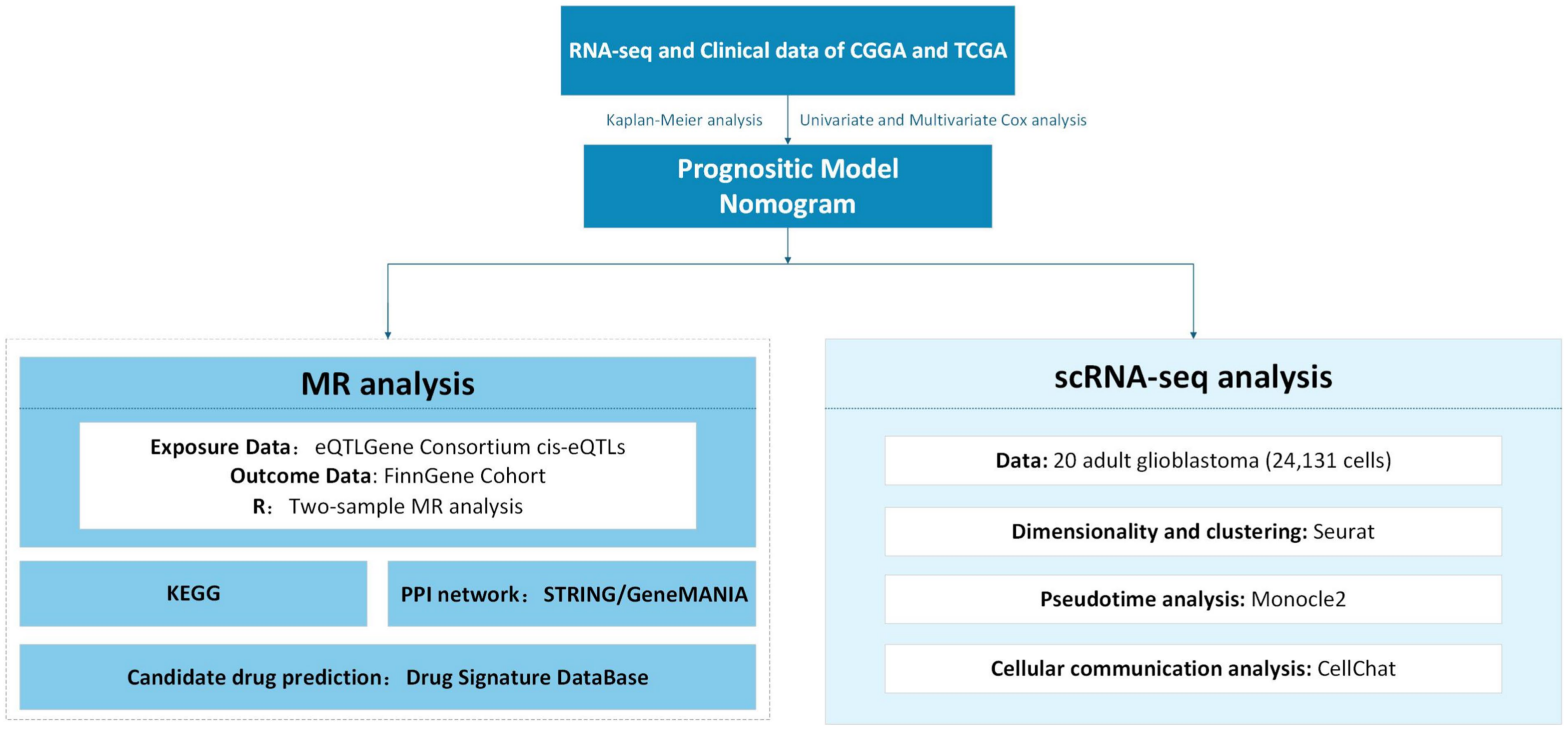
Integrative multi-omics analysis workflow for glioma prognostic modeling and translational medicine.

Mendelian randomization (MR) analyses were performed using a random-effects inverse variance weighted (IVW) method to estimate the causal effect of CD9 on glioma. Sensitivity analyses included weighted median (WM) estimation to validate robustness. Heterogeneity of individual SNP effects was assessed using Cochran’s Q test. Horizontal pleiotropy was evaluated via MR-Egger intercept analysis and the MR pleiotropy residual sum and outlier (MR-PRESSO) test to detect and remove outliers. A leave-one-out analysis was conducted to examine the influence of individual SNPs on overall estimates. The KEGG enrichment analysis of differentially expressed genes (DEGs) between CD9 high- and low-expression groups was performed using the ClusterProfiler R package. The study employed the Seurat (23) R package to investigate the distribution and dynamics of CD9 across different cell types using single-cell data from 24,131 cells derived from 20 adult glioblastoma samples. First, data normalization was performed to identify highly variable genes across cells. The uniform manifold approximation and projection (UMAP) algorithm was then applied for nonlinear dimensionality reduction. Cell annotation and clustering were conducted based on marker genes for each cell cluster, following the methodology described by Nefel et al. (24). Finally, CD9 gene expression levels were identified and localized across distinct glioma cell subpopulations at the single-cell resolution. Comprehensive methodological details are illustrated in the integrative flowchart provided in Figure 1.

## 3 Results

### 3.1 Association between CD9 expression levels and glioma prognosis

**Figure 2:**
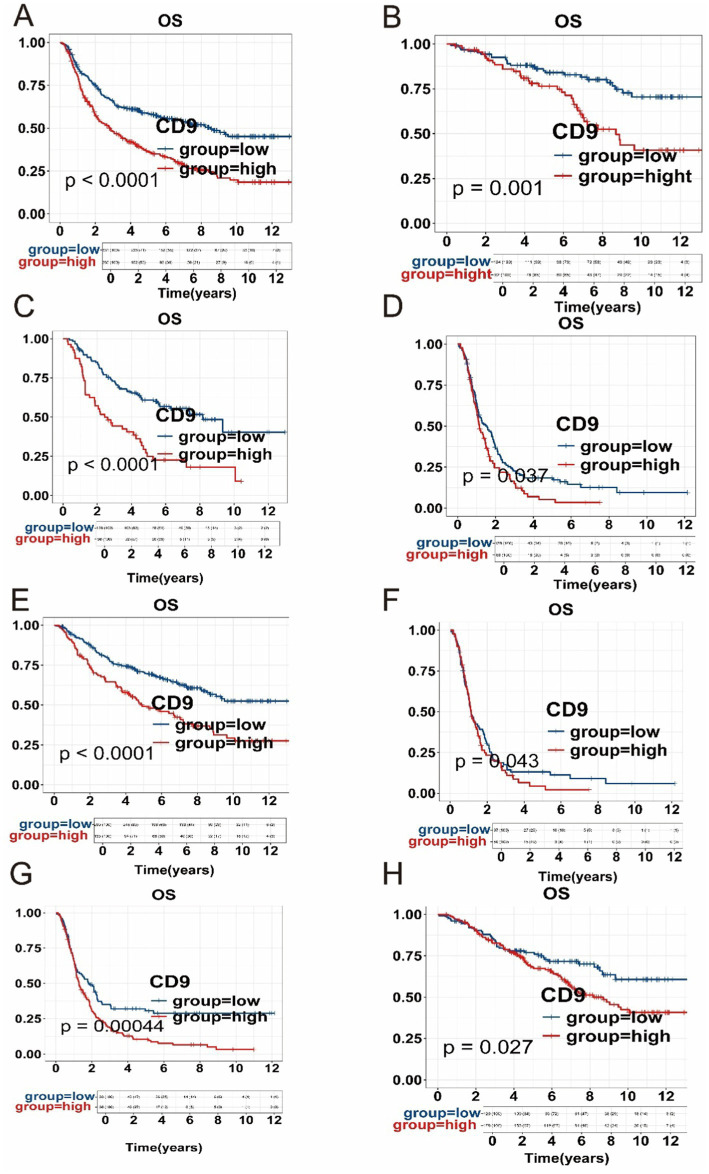
Kaplan–Meier (KM) survival curves. **(A)** Impact of CD9 expression on prognosis in the entire glioma cohort from the CGGA dataset. **(B)** Prognostic association of CD9 expression in grade 2 gliomas. **(C)** Prognostic association of CD9 expression in grade 3 gliomas. **(D)** Prognostic association of CD9 expression in grade 4 gliomas. **(E)** Relationship between CD9 expression and survival in the astrocytomas. **(F)** Relationship between CD9 expression and survival in the glioblastomas. **(G)** Relationship between CD9 expression and survival in the IDH mutant -type subgroup. **(H)** Relationship between CD9 expression and survival in the IDH wild-type subgroup.

The results presented in Figure 2 demonstrate that high CD9 expression is significantly associated with poorer overall survival across different WHO grades (*p* < 0.05). This adverse prognostic effect of elevated CD9 expression was consistently observed in both IDH-mutant and IDH-wildtype gliomas. Furthermore, within the astrocytoma and glioblastoma subgroups, high CD9 expression remained significantly correlated with worse outcomes. These findings highlight the robust prognostic value of CD9 expression across various glioma subtypes.

### 3.2 CD9 as an independent prognostic factor for overall survival in glioma

Univariate and multivariate Cox regression analyses were performed to evaluate the impact of Radiotherapy, Chemotherapy, WHO grade, IDH status, MGMT methylation status, 1p19q codeletion status, age, gender, and CD9 expression on glioma prognosis. The results demonstrated that, except for gender, all other variables were identified as independent prognostic factors. In the multivariate Cox regression model, high CD9 expression was associated with a hazard ratio (HR) of 1.28 (95% confidence interval [CI]: 1.03–1.58), indicating that elevated CD9 expression serves as a significant risk factor for glioma (*p* < 0.026) (Table 1).

**Table 1:** Univariate and multivariate Cox regression analyses.

Variables	Univariate					Multivariate				
	*β*	S.E	*Z*	*p*	HR (95%CI)	*β*	S.E	*Z*	*p*	HR (95%CI)
Grade										
WHO II					1.00 (Reference)					1.00 (Reference)
WHO III	1.03	0.14	7.52	**<0.001**	2.79 (2.14 ~ 3.65)	1.09	0.16	6.77	**<0.001**	2.97 (2.17 ~ 4.07)
WHO IV	2.41	0.13	18.46	**<0.001**	11.08 (8.58 ~ 14.31)	1.65	0.18	9.00	**<0.001**	5.18 (3.62 ~ 7.41)
IDH										
Mutant					1.00 (Reference)					1.00 (Reference)
Wildtype	1.80	0.10	18.62	**<0.001**	6.08 (5.03 ~ 7.35)	0.68	0.15	4.59	**<0.001**	1.97 (1.48 ~ 2.64)
MGMT										
Methylated					1.00 (Reference)					1.00 (Reference)
Un-methylated	0.61	0.09	6.52	**<0.001**	1.84 (1.53 ~ 2.21)	0.28	0.11	2.65	**0.008**	1.33 (1.08 ~ 1.64)
X1p19q										
Codel					1.00 (Reference)					1.00 (Reference)
Non-codel	1.85	0.17	10.88	**<0.001**	6.36 (4.56 ~ 8.88)	1.07	0.20	5.34	**<0.001**	2.90 (1.96 ~ 4.29)
Gender										
Female					1.00 (Reference)					1.00 (Reference)
Male	0.06	0.09	0.63	0.529	1.06 (0.89 ~ 1.26)	0.03	0.10	0.25	0.802	1.03 (0.84 ~ 1.26)
Group										
Low					1.00 (Reference)					1.00 (Reference)
High	0.60	0.09	6.61	**<0.001**	1.83 (1.53 ~ 2.19)	0.24	0.11	2.22	**0.026**	1.28 (1.03 ~ 1.58)
Radiotherapy										
0					1.00 (Reference)					1.00 (Reference)
1	0.24	0.12	2.03	**0.042**	1.27 (1.01 ~ 1.60)	−0.43	0.14	−3.00	**0.003**	0.65 (0.49 ~ 0.86)
Chemotherapy										
0					1.00 (Reference)					1.00 (Reference)
1	0.25	0.10	2.62	**0.009**	1.29 (1.07 ~ 1.56)	−0.50	0.12	−4.07	**<0.001**	0.60 (0.47 ~ 0.77)
Age	0.05	0.00	15.20	**<0.001**	1.06 (1.05 ~ 1.06)	0.03	0.00	5.91	**<0.001**	1.03 (1.02 ~ 1.04)

Bold values indicate statistically significant factors associated with glioma survival (*p* < 0.05).

### 3.3 Construction and validation of a CD9-associated glioma nomogram

**Figure 3:**
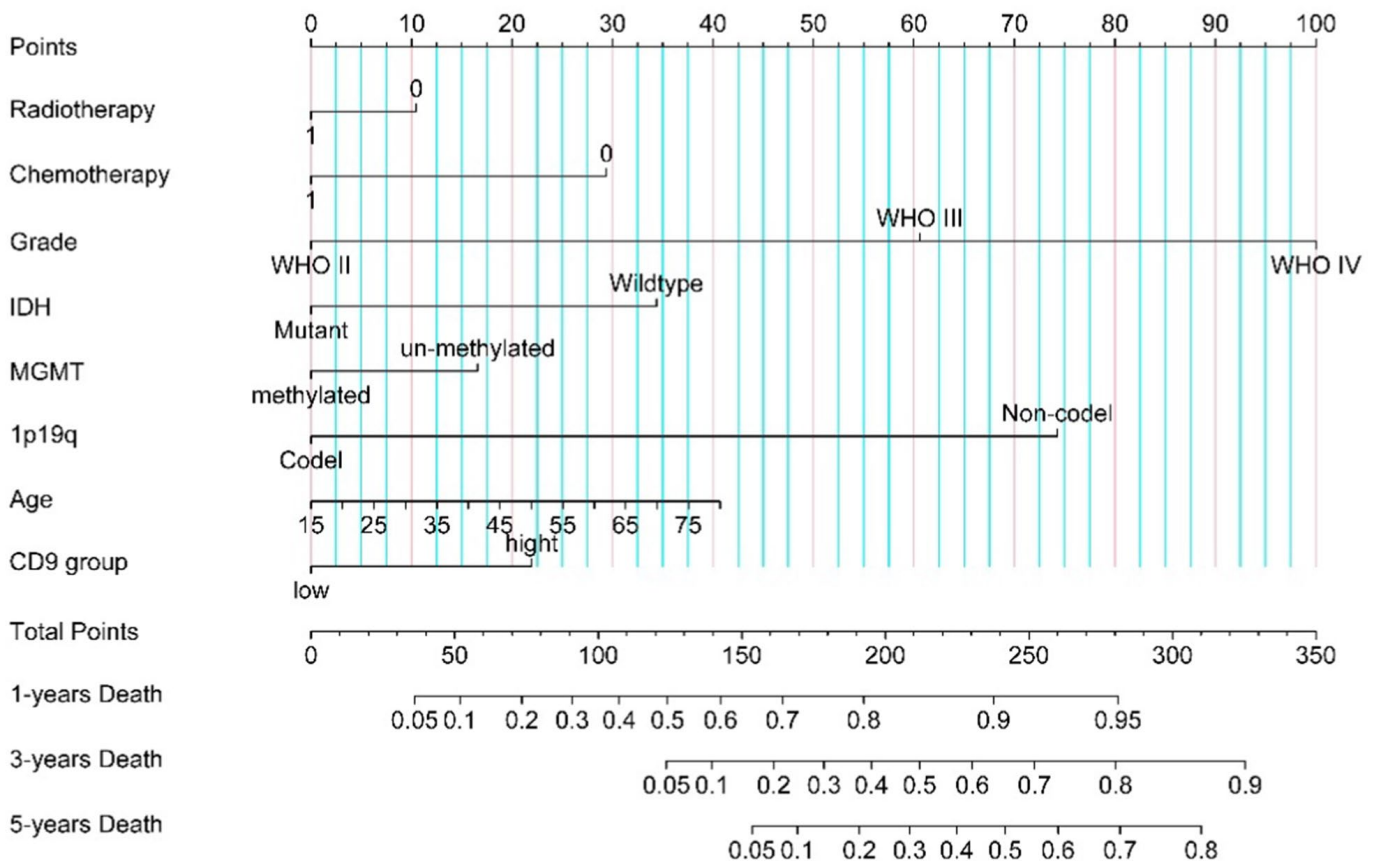
CD9-associated glioma nomogram.

The nomogram was developed based on multivariate Cox analysis results to predict 1-, 3-, and 5-year overall survival probabilities in glioma patients (Figure 3). To estimate survival probabilities, individual scores for each predictor (e.g., CD9 expression, WHO grade) were first assigned according to their respective values using the point scale at the top of the nomogram. Next, the scores for all predictors were summed to calculate a total score. Finally, the total score (shown on the bottom scale) was aligned with the corresponding survival probability scales to determine the predicted 1-, 3-, and 5-year survival rates. A hypothetical patient received radiotherapy (score: 0), underwent chemotherapy (score: 0), was diagnosed with WHO Grade III glioma (score: 60.5), had IDH wildtype status (score: 31.9), MGMT promoter methylation (score: 0), 1p/19q codeletion (score: 0), age 55 years (score: 24), and high CD9 expression (>236.92, score: 21.9). The total score for this patient was 138.3. According to the nomogram, the estimated probabilities of death at 1, 3, and 5 years are approximately 52, <10, and <5%, respectively.

**Figure 4:**
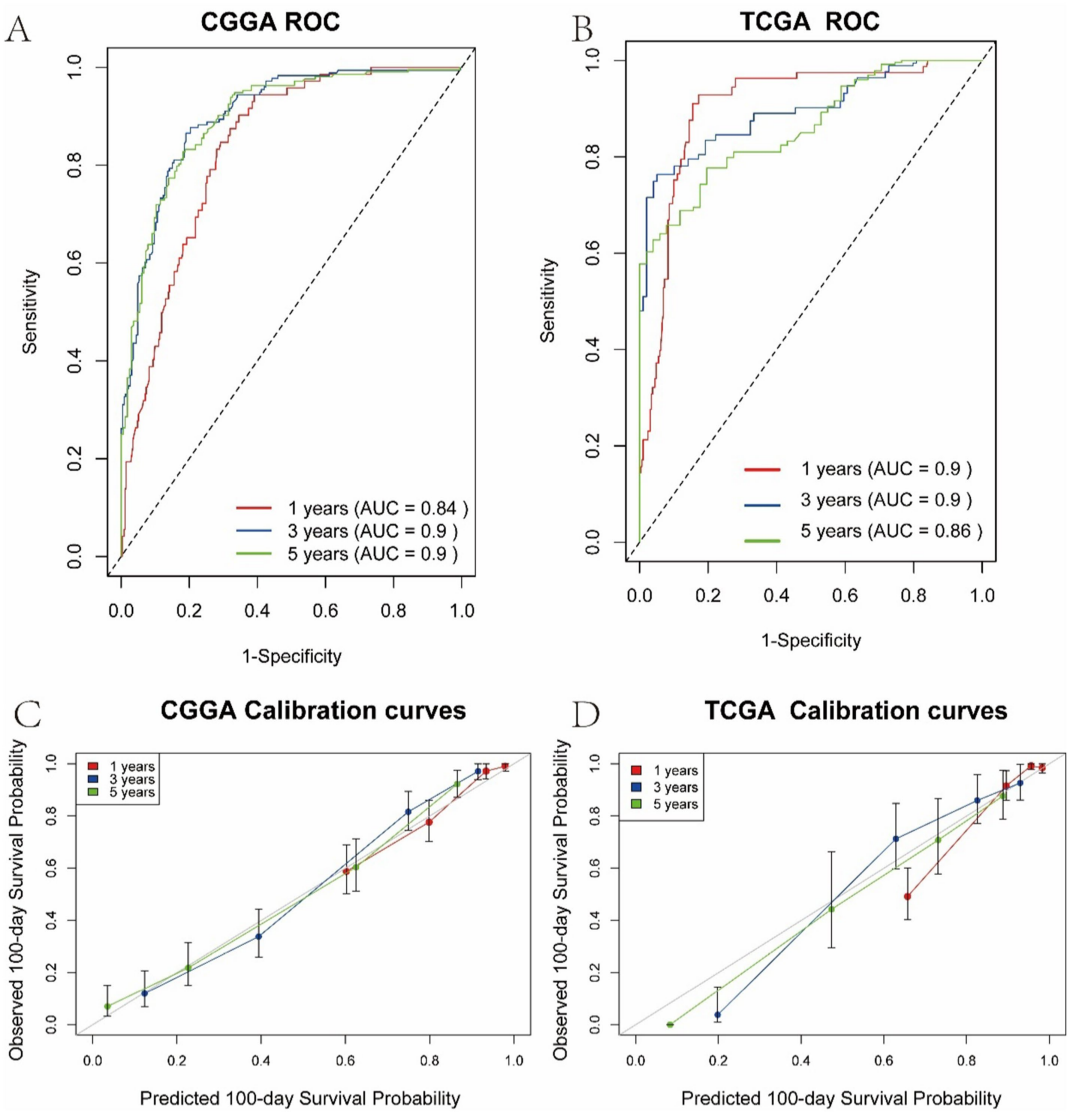
Calibration curves of the multivariate Cox regression model. **(A)** 1-, 3-, and 5-year ROC curves in the CGGA training cohort. **(B)** 1-, 3-, and 5-year ROC curves in the TCGA validation cohort. **(C)** Calibration curves for 1-, 3-, and 5-year survival predictions in the CGGA cohort. **(D)** Calibration curves for 1-, 3-, and 5-year survival predictions in the TCGA cohort.

Calibration curves generated via bootstrap resampling are presented in Figure 4. Figures 4A,C illustrate the 1-, 3-, and 5-year ROC and calibration curves for the training set (CGGA cohort), while Figures 4B,D show the corresponding results for the test set (TCGA cohort). In the CGGA cohort, the model-predicted survival probabilities achieved AUCs of 0.84, 0.90, and 0.90 for 1-, 3-, and 5-year survival, respectively, indicating strong concordance between predicted and observed outcomes. External validation using the TCGA cohort further demonstrated robust predictive performance, with AUCs of 0.90, 0.90, and 0.86 for the same time points, supporting the model’s generalizability. Additionally, the concordance index (C-index) was 0.805 ± 0.01 in the CGGA cohort and 0.859 ± 0.02 in the TCGA cohort. These results highlight the model’s accuracy in predicting survival probabilities and its applicability across both short- and long-term follow-up periods.

### 3.4 Mendelian randomization analysis

**Figure 5:**
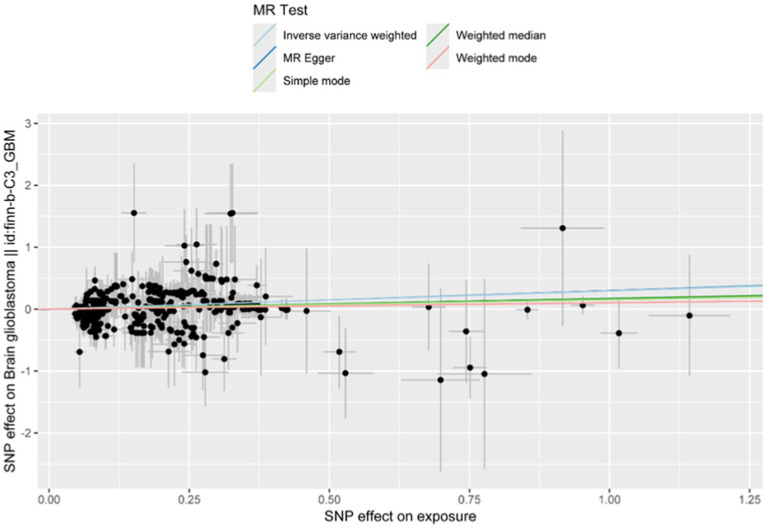
Mendelian randomization analysis using inverse variance weighted, MR-Egger, and weighted median methods scatter plot shows CD9’s effect on glioblastoma. Three lines represent effect size estimates calculated using inverse variance weighted (IVW), MR-Egger, and weighted median (WM) methods.

**Figure 6:**
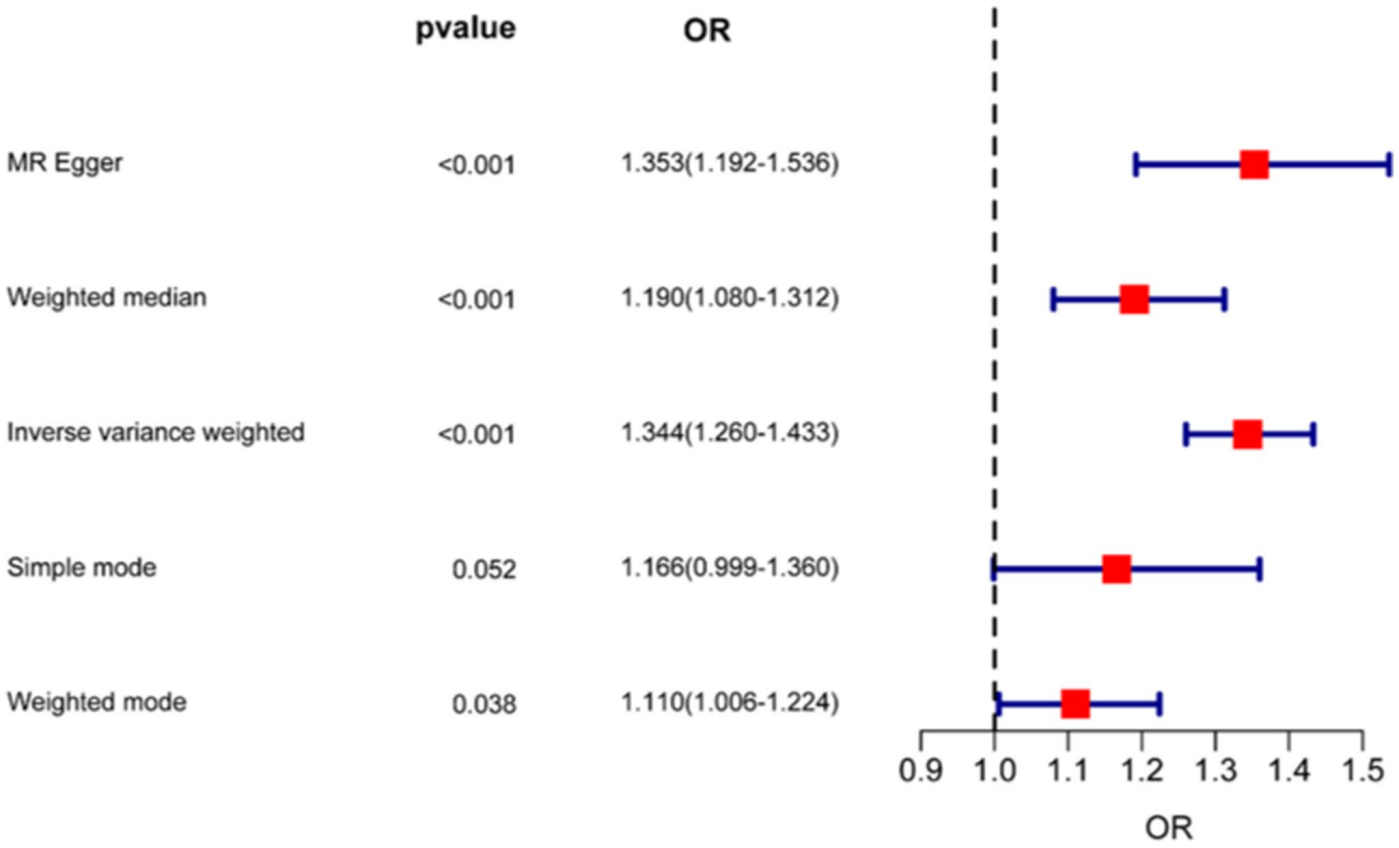
Forest plot of Mendelian randomization analysis estimating CD9-associated risk effects on glioma.

As shown in Figures 5, 6, the forest plot illustrates the effect estimates (odds ratio [OR]), 95% confidence intervals (CI), and corresponding *p*-values derived from the Mendelian randomization (MR) analysis of the CD9 gene. Various MR methods—including inverse variance weighted (IVW), weighted median (WM), and MR-Egger—were applied to assess the magnitude and direction of the association between CD9 expression and glioblastoma (GBM) risk. While some heterogeneity was observed across methods, all consistently supported a significant positive association between CD9 and increased GBM risk. Notably, the IVW method yielded an OR of 1.332 (95% CI: 1.250–1.420) per unit increase in CD9 expression. These findings reinforce the causal role of CD9 in gliomagenesis and progression and provide a theoretical rationale for targeting CD9 in GBM therapy.

### 3.5 KEGG analysis of CD9 high expression

**Figure 7:**
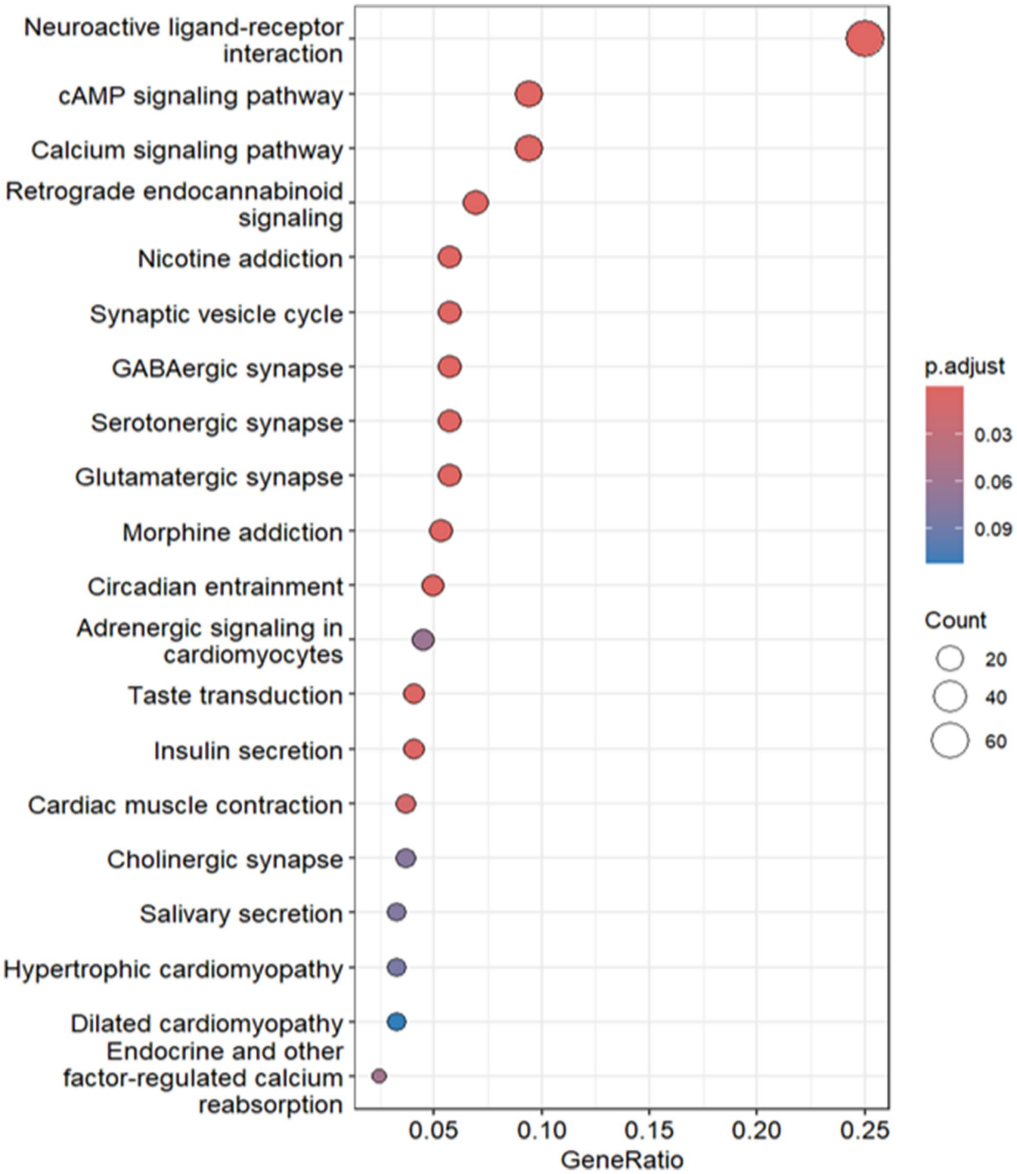
KEGG functional enrichment analysis of CD9 high expression.

As shown in Figure 7, a total of 244 genes were identified as being associated with high CD9 expression, while 399 genes were linked to low CD9 expression. Kyoto Encyclopedia of Genes and Genomes (KEGG) pathway enrichment analysis was conducted to elucidate the underlying biological functions and signaling pathways associated with these gene sets.

For genes linked to high CD9 expression, significant enrichment was observed in glioma-related pathways. The calcium signaling pathway (hsa04020) contained 23 genes (*p* = 8.81 × 10^−7^), including key receptors such as SLC8A2, HTR2C, GRIN1, CACNA1I, and GRIN2B, which regulate intracellular calcium levels and tumor microenvironment dynamics via glutamate interactions. Aberrant activation of this pathway may promote glioma proliferation, migration, and invasion through pro-growth signaling, suggesting therapeutic targets (25). The synaptic vesicle cycle pathway (hsa04721) included 14 genes (*p* = 3.97 × 10^−8^), such as STXBP1, RAB3A, RIMS1, CPLX2, and DNM1. Experimental evidence indicates that AMPAR-mediated synaptic transmission enhances glioma growth, with neuron-glioma co-cultures demonstrating increased tumor proliferation, implicating synaptic signaling in glioma progression (26). The GABAergic synapse pathway (hsa04727) involved 14 genes (*p* < 0.05), including GAD2, GNG3, GNG13, SLC12A5, and PRKCG. GABAergic interneurons critically drive H3K27M-mutant diffuse midline glioma (DMG) pathogenesis, as stimulating their activity in awake mice accelerates transplanted DMG cell proliferation (27).

For genes linked to low CD9 expression, enrichment was prominent in the cytokine-cytokine receptor interaction (hsa04060), complement and coagulation cascades (hsa04610), and viral cytokine receptor interaction (hsa04061) pathways. The cytokine-cytokine receptor pathway featured 35 genes (*p* = 3.79 × 10^−7^), such as CCL5, CXCL10, and IFNG, indicating impaired chemokine-mediated immune cell recruitment (e.g., CD8^+^ T cells and macrophages) in the immunosuppressive glioma microenvironment (28). CCL5 and CCL12 were further implicated in tumor proliferation and invasion [The Classical Hodgkin’s Lymphoma Microenvironment and Its Role in Promoting Tumor Growth and Immune Escape (29)]. In the viral cytokine receptor pathway, upregulated genes (*p* = 1.39 × 10^−5^), including TNFRSF1A, CCL21, IL2RB, IL22RA1, and XCR1, suggest that oncolytic viruses exploit tumor-specific immune evasion mechanisms to selectively lyse tumor cells, activating anti-tumor immunity via DAMPs and PAMPs (30).

### 3.6 Single-cell analysis

**Figure 8:**
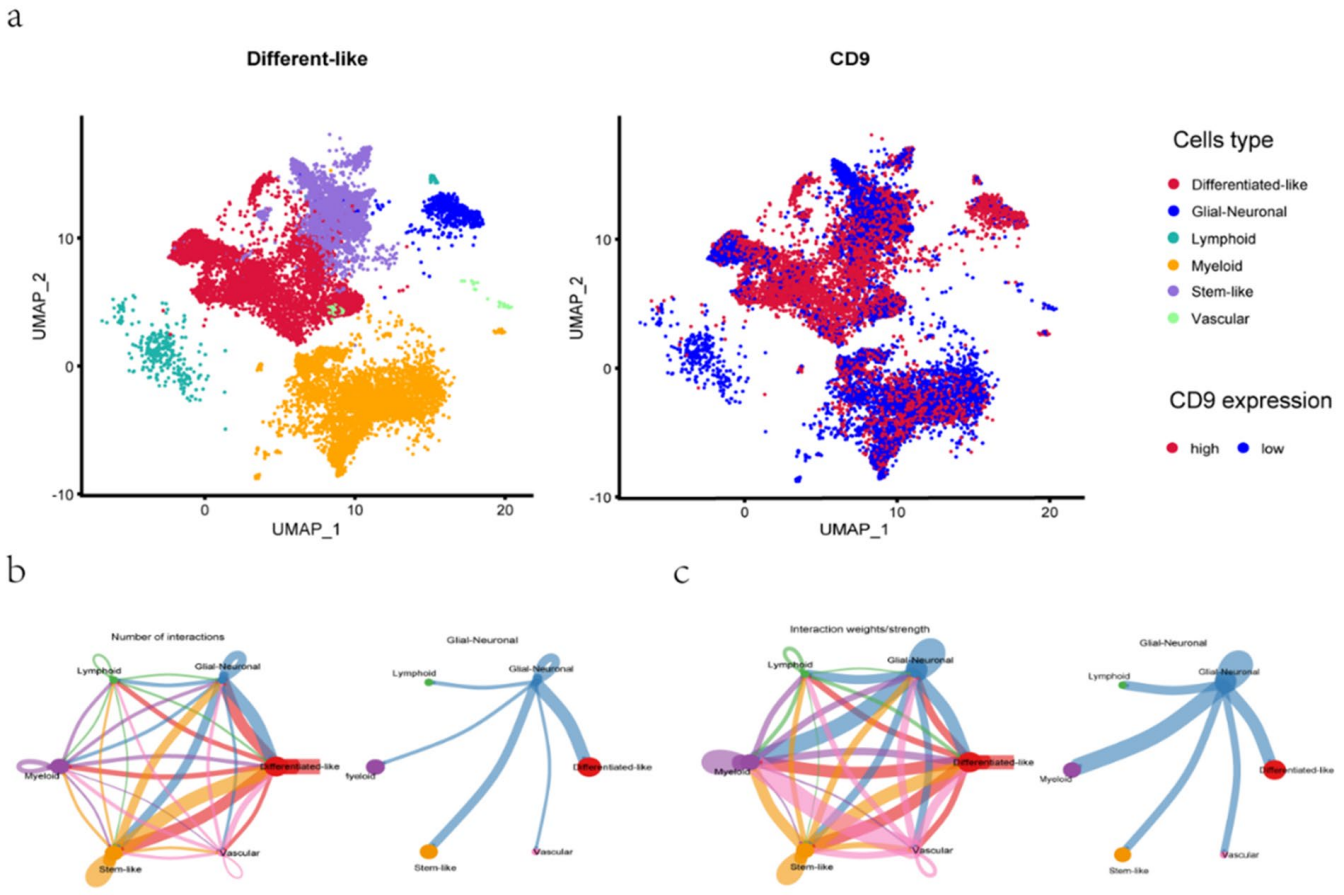
UMAP dimensionality reduction and cell–cell communication analysis. **a**: UMAP for the dimension reduction and visualization of 6 cell types (left) and UMAP for the dimension reduction and visualization of cells with high or low CD9 expression (right). **b-c**: **(b)** The number of interactions and **(c)** the interaction weights/strengths between cells. The color and width of the lines represent the number of interacting pairs between cell types.

Following quality control of single-cell RNA sequencing (scRNA-seq) data, 24,131 high-quality cells were retained for downstream analysis. UMAP in Figure 8 was employed to cluster cells based on gene expression variability, with distinct cell types color-coded: differentiated-like cells, glial-neuronal cells, lymphoid cells, myeloid cells, stem-like cells, and vascular-associated cells. Differentiated-like and myeloid cells exhibited significant heterogeneity, forming multiple subclusters, whereas vascular-associated cells were the least abundant. These findings collectively highlight the pronounced cellular heterogeneity in gliomas, primarily driven by differentiated-like and myeloid cell populations.

**Figure 9:**
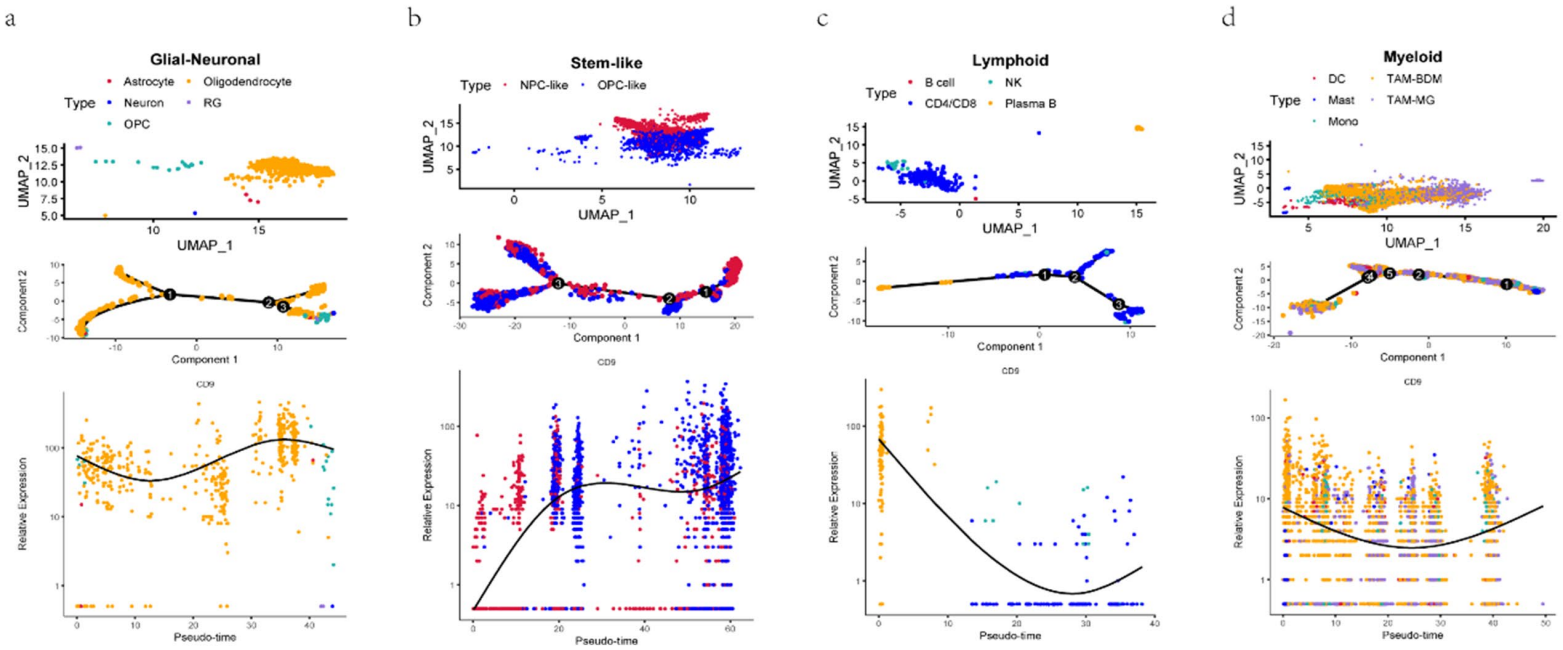
Pseudotemporal trajectory analysis of glioma cellular differentiation. **a–d**:Cell subpopulations (upper panels), single-cell motility trajectories (middle panels), and dynamic changes in CD expression during cell differentiation (lower panels) are visualized for Glial-neuronal. **(a)**, Stem-like **(b)**, Lymphoid **(c)**, and Myeloid **(d)** cells. UMAP plots show the main subclusters of cells. The “Monocle2” algorithm was used to generate trajectory plots for cells and the dynamic changes in CD9 expression. Each dot represents a single cell colored according to its cluster, and the solid black line in the lower panels shows the LOESS fit.

Pseudotemporal trajectory analysis in Figure 9 revealed the differentiation dynamics of glioma stem-like cells. Neural progenitor-like (NPC-like) cells (31), characterized by self-renewal capacity and potential to differentiate into neurons, astrocytes, or oligodendrocytes, were predominantly localized to the right side of the trajectory. Along the differentiation axis (leftward), NPC-like cells with high CD9 expression transitioned toward oligodendrocyte precursor-like (OPC-like) cells with low CD9 expression. OPC-like cells, marked by genes such as PDGFRα and NG2, represent an intermediate state with migratory and proliferative capabilities before terminal differentiation into myelinating oligodendrocytes. This trajectory suggests that CD9 may regulate tumor proliferation and migration by promoting the transition from NPC-like to OPC-like phenotypes. The analysis further confirmed diverse phenotypic states within glioma stem-like cells, each defined by unique gene expression profiles, and underscored dynamic CD9 expression patterns across distinct biological differentiation processes in glioma.

### 3.7 Protein–protein interaction (PPI) network

**Figure 10:**
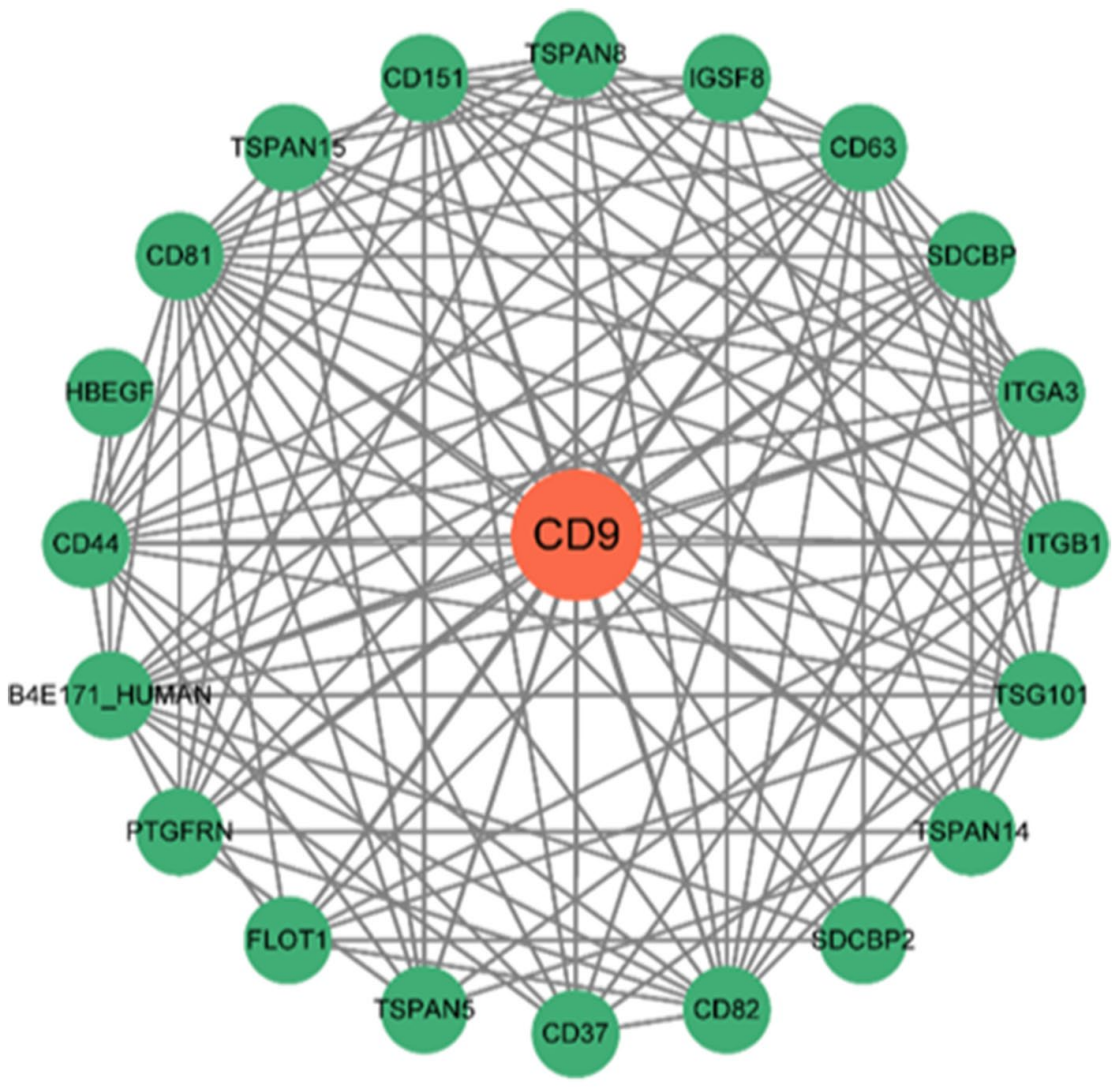
PPI network constructed using the STRING database. The PPI network illustrates interactions between CD9 and other genes or proteins, where nodes represent genes/proteins and edges represent interactions.

The network comprises 20 nodes (Figure 10), revealing complex interactions between CD9 and other genes or proteins that may participate in glioma biology. These interactions provide critical network-based biological insights for further exploration of gene functions and therapeutic target development.

The figure displays interactions between CD9 and 20 additional genes, including interaction types (e.g., physical interactions, co-expression, shared protein domains) and functional pathway annotations.

**Figure 11:**
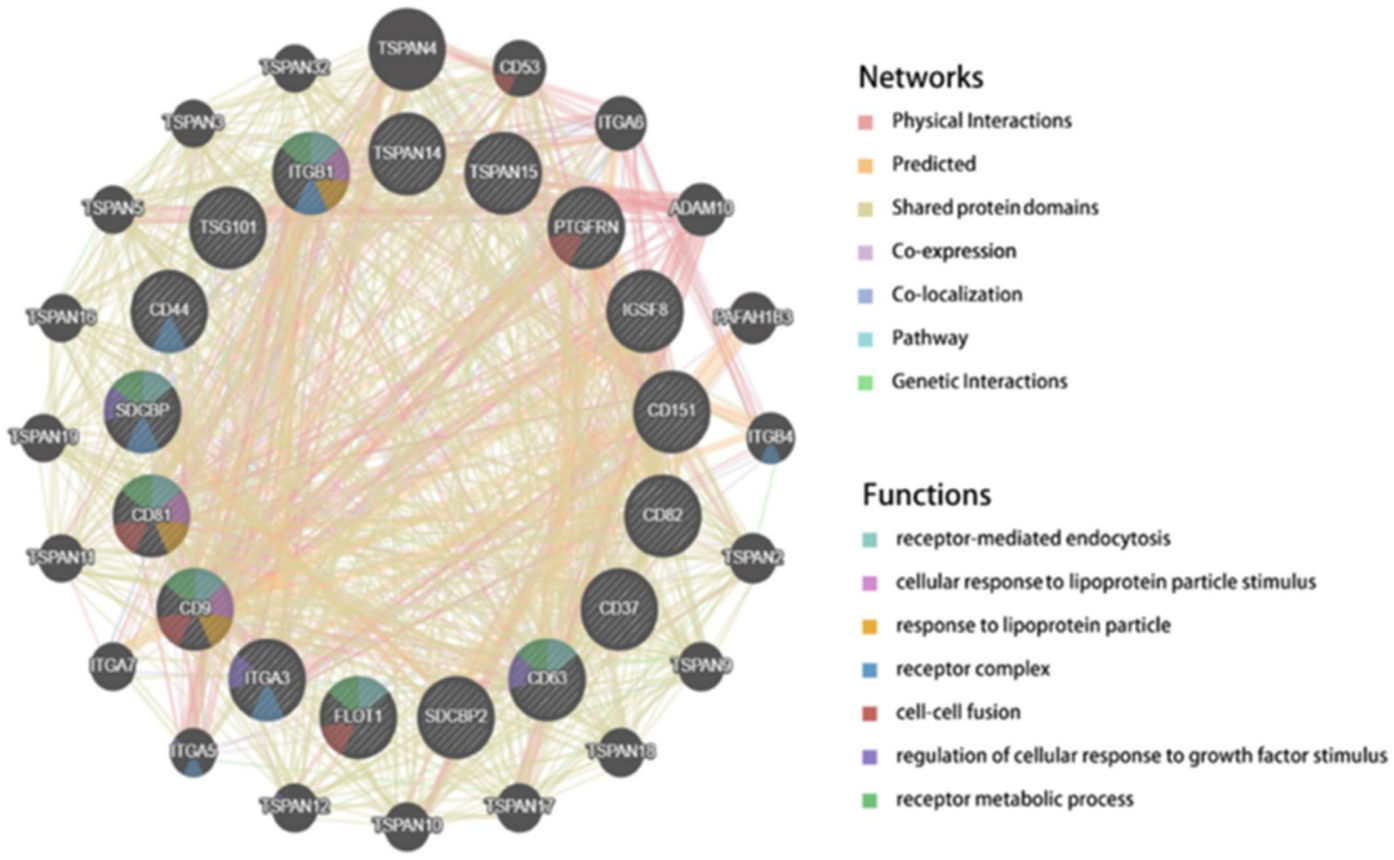
GeneMANIA-constructed PPI network.

The GeneMANIA-derived PPI network^3^ includes 20 potential interacting genes (Figure 11). Interaction types are categorized as follows: 43.82% physical interactions, 26.56% predicted interactions, 19.32% shared protein domains, 6.92% co-expression, and 2.09% co-localization. The highly dense intercellular connections within the PPI network suggest that CD9 may contribute to sustained enhancement of glioma cell proliferation signaling pathways.

### 3.8 Prediction of potential drug targets using DsigDB

Table 2 presents candidate biomarkers predicted by DsigDB for potential drug targets. Among these, emetine MCF7 DOWN was the most significant drug associated with CD9. Previous studies have reported that emetine induces glioblastoma cell deat (23). Phenome-wide association study (PheWAS) analysis at the gene level revealed no significant associations between CD9 and other traits, indicating high specificity of CD9. These findings suggest that CD9 is a promising therapeutic target in glioma.

**Table 2:** PPI network-related drug targets and associated genes.

Term	*p*-value	Adjusted *p*-value	Genes
Puromycin aminonucleoside	<0.001	0.02	ITGB1; ITGA3
Etodolac	<0.001	0.03	ITGB1; CD44
Nocodazole	<0.001	0.03	ITGB1; SDCBP; FLOT1; CD44
ML-7	<0.001	0.03	ITGB1; ITGA3
Carmustine	<0.001	0.03	ITGB1; CD151; ITGA3; CD44
LAMININ BOSS	<0.001	0.04	ITGB1; CD151; CD44
PHENCYCLIDINE	<0.001	0.04	CD63; CD81; CD44
Cyclohexanecarboxamide	<0.001	0.04	ITGB1; ITGA3
Emetine MCF7 DOWN	<0.001	0.04	ITGB1; CD9; CD44
Scriptaid	<0.001	0.04	ITGB1; CD82
Electrocorundum	<0.001	0.04	ITGB1; ITGA3

This table displays candidate drugs predicted by the Drug Signature Database (DsigDB) to be associated with potential therapeutic targets. Results include drug names, *p*-values, adjusted *p*-values (e.g., FDR-corrected), and related gene information to evaluate potential drug-target interactions.

## 4 Discussion

Our analysis of 1,033 glioma patients demonstrated a strong correlation between elevated CD9 expression and poor prognosis, with high CD9 expression associated with significantly shorter overall survival compared to low expression. This positions CD9 as a pivotal prognostic biomarker in glioma, offering independent prognostic value beyond established factors such as age, WHO grade, IDH mutation, and 1p19q codeletion status in multivariate Cox analysis. These findings align with previous studies highlighting tetraspanins in cancer biology (10), yet extend the understanding by integrating CD9 into a multifactorial survival model, thereby enriching insights into glioma progression.

Single-cell sequencing revealed enriched CD9 expression in neural progenitor-like (NPC-like) glioma stem cell subpopulations, with pseudotemporal trajectory analysis showing progressive downregulation during differentiation toward oligodendrocyte precursor-like (OPC-like) cells. This aligns with the stemness maintenance theory, where NPC-like cells contribute to therapy resistance and recurrence (31). Notably, CD9 exhibited differential expression in tumor-associated macrophages (TAMs), and CellChat-predicted enhanced TGF-*β* signaling suggests its role in modulating tumor-immune crosstalk, warranting validation via spatial transcriptomics or multiplex immunofluorescence. Protein–protein interaction (PPI) network analyses and DsigDB drug predictions further identified CD9 as a therapeutic target, with emetine emerging as a promising candidate due to its reported anti-glioblastoma activity.

While existing glioma prognostic models predominantly rely on clinicopathological features (24), our CD9-integrated model synergizes traditional and molecular biomarkers, achieving superior predictive accuracy. This advancement supports personalized treatment strategies, enabling risk stratification for aggressive therapies (e.g., CD9-targeted agents) in high-risk patients and conservative management in low-risk cohorts. Moreover, MR established the causal relationship between CD9 and glioma by integrating SNPs associated with CD9 and glioma GWAS data. To our knowledge, this study represents one of the first to employ MR in identifying potential therapeutic targets for glioma, leveraging large-scale, publicly available glioma GWAS datasets. By implementing MR analyses through the FinnGen dataset, we mitigated confounding biases inherent in observational measurements, thereby confirming the causal link between CD9 and glioma. This approach provides robust causal evidence to inform subsequent mechanistic and translational investigations. Single-cell sequencing further underscores its utility in dissecting cellular heterogeneity and developmental trajectories, as demonstrated by our cell subpopulation annotation guided by marker genes defined by Neftel et al. (32), which clarified CD9’s role in promoting tumor cell states.

Although this study primarily focuses on the prognostic value of CD9 in glioma, the exploration of its underlying mechanisms provides deeper insights into glioma biology. CD9, a transmembrane protein, has been reported to regulate tumor cell proliferation, apoptosis, migration, and invasion in various cancers (24, 33). Notably, CD9 is preferentially expressed in glioblastoma stem cells (GSCs) and is indispensable for maintaining their self-renewal capacity (34). Co-immunoprecipitation and immunofluorescence analyses revealed that CD9 stabilizes the interleukin-6 (IL-6) receptor subunit gp130 by preventing its lysosomal ubiquitination-mediated degradation, thereby activating the BMX-STAT3 signaling pathway to sustain GSC stemness and tumorigenicity (35). This finding offers critical insights into CD9’s role in glioma pathogenesis. In glioblastoma, CD9 may further promote tumor cell migration and invasion by participating in extracellular vesicle-mediated processed (36). Additionally, CD9’s association with GSC properties, including self-renewal and chemoresistance (37), underscores the need for further mechanistic studies. Future research should delineate CD9’s signaling networks and molecular interactions (38), providing a theoretical foundation for developing CD9-targeted therapies.

KEGG enrichment analysis revealed that CD9 high-expression-associated genes were significantly enriched in the calcium signaling pathway (*p* = 8.81 × 10^−7^), synaptic vesicle cycle pathway (*p* = 3.97 × 10^−8^), and GABAergic synapse pathway (*p* = 1.39 × 10^−5^). Dysregulated calcium signaling may drive glioma proliferation and invasion by modulating glutamate release in the tumor microenvironment (25), while synaptic pathway enrichment underscores the critical role of neuron-glioma interactions in tumor progression, consistent with recent evidence that neuronal activity directly stimulates glioma growth (29). Conversely, CD9 low-expression-associated genes were enriched in immune-regulatory pathways, such as cytokine-cytokine receptor interactions (*p* = 3.79 × 10^−7^), suggesting that CD9 may suppress chemokine-mediated immune cell infiltration (e.g., CCL5, CXCL10) to shape an immunosuppressive microenvironment (28). This dual role of CD9 highlights its potential to drive gliomagenesis through both pro-oncogenic signaling and immune evasion mechanisms.

This study’s retrospective design, though mitigated by robust statistical methods, necessitates prospective validation. While CGGA and TCGA datasets provide broad coverage, expanding to multiethnic cohorts and incorporating normal tissue controls would enhance generalizability. Furthermore, the eQTLGen used in this study included individuals of non-European ancestry, whereas the glioma GWAS data were derived from European populations. This population heterogeneity may introduce potential bias in MR effect estimates. The limited single-cell sample size (*n* = 20) and absence of spatial resolution constrain tumor-specific expression profiling. Future work should integrate multi-omics data and combine CD9 with emerging biomarkers to refine prognostic models. Mechanistic exploration of CD9’s role in GSC-TAM crosstalk and extracellular vesicle biology, alongside preclinical testing of CD9-targeted therapies (39), remains critical to advancing glioma therapeutics.

## 5 Conclusion

This study developed and validated a CD9-based prognostic model for glioma, with MR analyses confirming the causal relationship between CD9 and gliomagenesis and progression. Single-cell analysis and PPI network exploration revealed the distribution patterns, dynamic expression trends, and interaction networks of CD9 across distinct glioma cell subpopulations. These findings suggest that CD9 may promote tumor proliferation and invasion by inducing differentiation of glioma stem cells. Drug prediction analysis further identified emetine as a potential CD9-targeting therapeutic agent. Collectively, this study systematically elucidates the central role of CD9 in glioma through prognostic modeling, molecular mechanism investigation, and therapeutic target prediction, providing a multifaceted framework for advancing glioma research and therapy.

## Data Availability

The original contributions presented in the study are included in the article/Supplementary material, further inquiries can be directed to the corresponding author.

## Footnotes

^1^ https://www.scielo.org.mx/scielo.php?pid=S1665-50442021000100022&script=sci_arttext

^2^ https://eqtlgen.org/

^3^ https://genemania.org/
