# Supplementary figures and images for: CD9 expression rivals IDH mutation as a prognostic marker in glioma: a novel nomogram approach
Source: Front Neurol. 2025 Apr 30;16:1507443. doi: 10.3389/fneur.2025.1507443 (PMC12096848; doi:10.3389/fneur.2025.1507443)

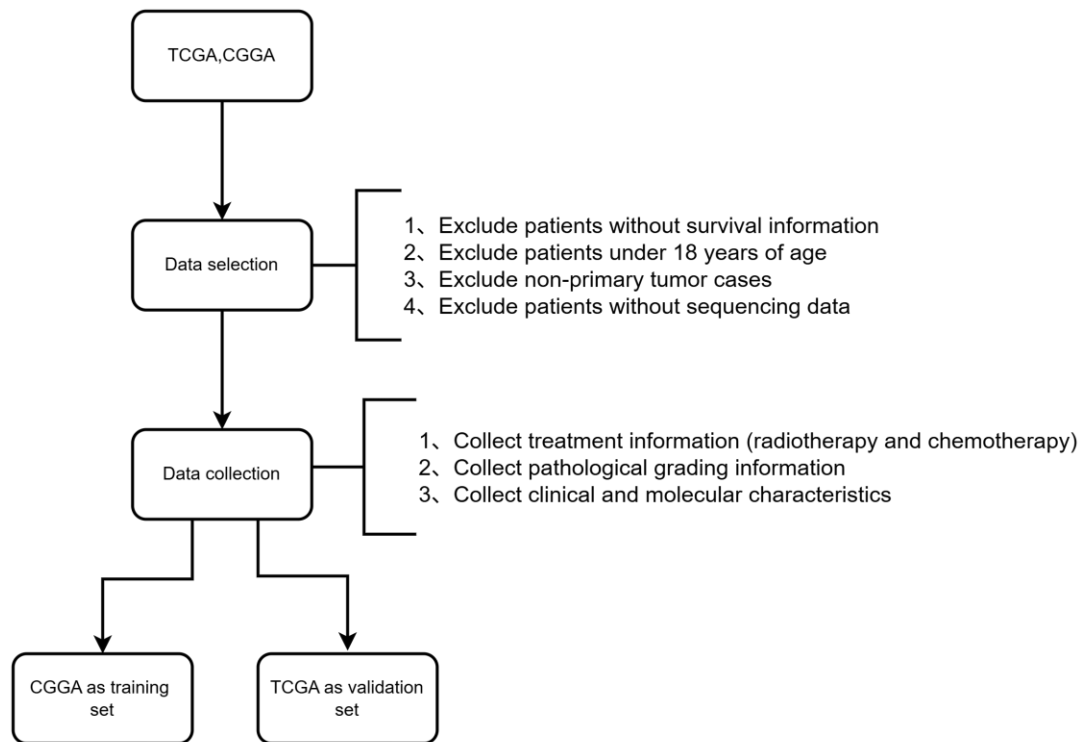

SFigure1. Flowchart of patient inclusion and exclusion criteria

Supplement: Supplementary file 1 [file Data_Sheet_1.pdf]
